# Bangladesh: 50 Years of Advances in Health and Challenges Ahead

**DOI:** 10.9745/GHSP-D-23-00419

**Published:** 2024-02-28

**Authors:** Henry B. Perry, Ahmed Mushtaque Raza Chowdhury

**Affiliations:** a Health Systems Program, Department of International Health, Johns Hopkins Bloomberg School of Public Health, Baltimore, MD, USA.; b James P. Grant School of Public Health, BRAC University, Dhaka, Bangladesh.; c Keough School of Global Affairs and Eck Institute of Global Health, University of Notre Dame, South Bend, IN, USA.

## Abstract

Bangladesh has inspired the rest of the world with its remarkable health achievements over the past half-century. A considerably stronger government investment in health care is now needed to achieve universal health coverage and “Health for All” in Bangladesh.

## Key Messages

- Bangladesh is a “positive deviant” as a result of its progress from being the second poorest country in the world to implementing world-class programs in family planning, immunizations, promotion of oral rehydration therapy for diarrhea, detection and treatment completion for TB, and much more.
- The recently published book, *50 Years of Bangladesh: Advances in Health,* highlights these and many other achievements and provides an overview of the daunting challenges that must be overcome in the next 50 years if Bangladesh is to achieve universal health coverage and “Health for All.”
- The most important of these challenges include major increases in government expenditures for health, building of a strong primary health care system that relies on a professionalized cadre of community health workers that reach every home on a regular basis, catastrophic health insurance for all citizens, innovations in health care delivery within the long-standing culture of reliance on research and evidence, and strong independent civil society engagement by groups such as Bangladesh Health Watch.

## INTRODUCTION

A colleague said many years ago, “Bangladesh is to development as Paris is to fashions.” One could also say that Bangladesh is a positive deviant—in a class by itself compared to other low-income countries in the achievements it has made in health and development since its independence in 1971. At that time, it was the second-poorest country in the world (after Upper Volta, now Burkina Faso). Now, Bangladesh is a lower-middle-income country,^1^ and the government believes that in 20 years, it will become a high-income country.

The reasons for this remarkable progress—from, as Henry Kissinger infamously called it in the early 1970s, “an international basket case,” to a country bustling with empowerment, optimism, and a “basket of innovations”—are complex. But among the important contributors is Bangladesh’s extraordinary achievement in improving the health of its people. This is the subject of a recently published book, *50 Years of Bangladesh: Advances in Health.*^2^ Convened by the Bangladesh Health Watch, an independent citizens’ oversight group,^3^ this book is a most fitting sequel to another book written by one of us (HBP) in 2000.^4^ The other of us (AMRC) is the principal editor of the recent book.

As one reflects on Bangladesh’s gains of the past half-century and anticipates the challenges and opportunities of the next half-century, now is an opportune moment to remember the 3 million Bangladeshis who lost their lives in the struggle for independence, the millions whose lives were cut short by death from readily preventable or treatable conditions, and the hundreds of millions who suffered from stigma, discrimination, and subhuman poverty. This is also an opportune moment to remember with gratitude those who provided fearless and inspired leadership in overcoming what to many would have been insurmountable odds. Many of these people are no longer with us today. Among the many hundreds if not thousands of people that one might mention, a special remembrance is owed to Bangladesh’s founder, Bangabandhu Sheikh Mujibur Rahman; the founder of BRAC, Sir F. H. Abed; the founder of Grameen Bank and Nobel Peace Prize laureate, Professor Muhammad Yunus; the freedom fighter, war surgeon, and founder of one of Bangladesh’s leading nongovernmental organizations (NGOs), Gonoshasthya Kendra; and the champion of Bangladesh’s renowned drug policy, Dr. Zafrullah Chowdhury.

After reading all 370 pages of this book, one could not, perhaps, help but stand in awe at the collective action required to produce this book—103 authors and co-authors working together to produce 20 chapters. And one cannot help but also stand once again in awe at the extraordinary achievements described in this book. Collectively, they are the product of so many individuals, programs, organizations, and institutions that dedicated themselves to improving the health and well-being of the 170 million people who now live in Bangladesh.

## HEALTH ACHIEVEMENTS IN BANGLADESH OF NOTE

In their 2013 series of articles in *The Lancet,* Mushtaque Chowdhury and colleagues referred to the “the Bangladesh paradox: exceptional health achievement despite economic poverty” that was achieved with gender- and equity-oriented, highly focused nationwide interventions.^5^ Among the many noteworthy achievements, at least a few that are well known to many are important to recall.

- The improvement in life expectancy at birth from 50 years in 1971 to 72 years in 2021^6^
- The reduction in mortality of children younger than 5 years of age from 251 in 1971 to 31 deaths per 1,000 live births in 2021^7^^,^^8^
- The reduction in the total fertility rate from 6.6 in 1975 to 2.0 births per woman in 2021^9^^,^^10^
- The expansion in coverage of childhood immunizations from 2% in 1978 to nearly 100% in 2021 as well as the elimination of polio^10^^,^^11^
- The discovery in Bangladesh of oral rehydration therapy as an effective treatment for dehydration produced by diarrhea and the implementation by BRAC of its national Oral Therapy Extension Program, setting the foundation for the highest usage rate (87%) in the world of oral rehydration therapy for childhood diarrhea^12^ and contributing to the decline in the percentage of deaths in children younger than 5 years from diarrhea from 25% in 1970 to 2% in 2000 and since^11^
- The detection of more than 70% of cases of pulmonary-active TB and the completion of treatment in 90% of detected cases^13^
- The reduction in the rate of open defecation from 34% to less than 1% between 2000 and 2015^12^
- The development of strategies that have mitigated the mortality and consequent sufferings associated with cyclones and floods through early warning systems, construction of shelters and housing that provide a safe haven, and pre-planning for rapid response when disaster strikes^14^

Routine home visits by women community health workers (CHWs) were responsible, to a considerable degree, for the remarkable progress in child and reproductive health. Female CHWs visited all homes to promote family planning at a time when women could not leave their homes or immediate neighborhoods. These workers visited all homes to mobilize mothers to take their children to special sites for immunizations on days when a government mobile team would arrive in their community. They visited more than 12 million homes specifically to teach them how to prepare and administer oral rehydration fluid to prevent fatal dehydration from diarrhea. These actions were all the result of community engagement and the empowerment of women as CHWs. As the book states^15^:

*The adeptness of NGOs to successfully transform ordinary, less educated rural women into confident and self-dependent individuals and inspiring such women to participate in social reforms has been a fundamental element in Bangladesh’s progress.*

The book concludes by declaring that^16^:

*[c]reating a cadre of female health workers …, demystifying health care and making it more understandable for the general public have been found to be critical factors for success of Bangladesh’s health sector.*

All of these achievements were aided and abetted by the benefits of broad socioeconomic development, of course. Bangladesh’s progress in women’s empowerment, elimination of illiteracy, raising the level of education (especially for girls), formation of women’s groups and microcredit programs, other poverty alleviation programs, and expansion of agricultural output, as well as improvements in roads, communications, and other infrastructure, have all helped to make these health achievements possible.

Bangladesh now stands at a critical moment in its history. It has a proud heritage of achievement in health, social development, and poverty alleviation. The achievements of the past 50 years have given Bangladesh a momentum that will propel it into ever-increasing economic prosperity. The unanswered question now is, “Will that economic prosperity also produce ‘Health for All’?” Will the poor enjoy the benefits now available to people who live in high-income countries, similar to the better-off Bangladeshis?

## THE WAY FORWARD

There are several key priorities for the next 50 years that arise from this book.

### 1. Support for Bold Innovations From “Outside the Box”

What has worked in the past is not necessarily going to work in the future. Bangladesh has picked many of the “low-hanging fruits” in its success in reducing child mortality and fertility. However, picking the “high-hanging fruits” by creating a strong and effective primary health care (PHC) system for all citizens will require a bold and innovative commitment of the government together with civil society—with considerably expanded resources and rigorous ongoing assessment of effectiveness. A second-generation PHC system is needed. As Amartya Sen has reminded us^17^:

*The key to Bangladesh’s laudable success has been the avoidance of the twin dangers of inertia and smugness. The future will demand more from these virtues.*

The country must work toward achieving universal health coverage, which, as we know, is achieved when all citizens, irrespective of their socioeconomic condition, can access quality health services they need without suffering financial hardship. Although the government has committed to achieving universal health coverage by 2030, it has not yet taken any visible concrete steps toward this end. Accountability of the health system is a major issue. An important first step in addressing this would be to set up an independent National Health Security Office. This body would be tasked to act as the financier of the health sector and would monitor government health expenditures through rigorous internal audits.

Governance is the key and much has been written about the poor management of the country’s health systems, both public and private. Absenteeism is the name of the game, and at any given time, not even two-thirds of the relevant staff are found in facilities that require their presence 24 hours a day/7 days a week. And so is corruption. We don’t think these management problems have been analyzed enough, nor have the root causes that have allowed these governance failures to happen and persist. The same goes with the private health care sector, where the lack of regulations and the failure to enforce the few regulations that do exist have made the private health care sector into an uncontrollable monster.

### 2. Major Expansion of Government Funding for the Health Sector

The government’s health expenditure as a percentage of the gross domestic product (GDP) is among the lowest in the world—0.7%.^18^ The poor absorptive capacity of the Ministry of Health leads to a large portion of the scarce resources going unused. The percentage of out-of-pocket expenditures is among the highest in the world (67%),^18^ causing 5 million people to become impoverished every year.^18^ Even worse, one-third of the money budgeted by the government for health is not actually spent.^18^ The share of public sector financing accounts for only 23% of Bangladesh’s total health expenditures, and this percentage has declined from 37% in 1997.^18^ Per capita total health expenditure is only US$37, half of what India spends.^18^ Expenditure of government health funds is inequitable, with poorer rural divisions receiving lower allocations per capita and government tertiary hospitals providing care disproportionately to better-off citizens.

Major expansions of government funding for the health sector will be required together with large increases in the size of the health workforce. This will require a tripling of the portion of the GDP that the government spends on health services, from 0.7% to 2.0%. Funding for PHC will need to be expanded. The private sector will continue to play an important role, particularly for the better-off section of the community, but it will need to be closely regulated.

### 3. Development of a Strong and Accountable Primary Health Care System

Although Bangladesh has made notable strides in the development of a strong, effective, and affordable PHC system, it needs to give greater emphasis on the prevention, early identification, and ongoing treatment of noncommunicable diseases, almost all of which are chronic. This will require, among other things, a major expansion of the number of PHC workers, distributed in a pyramid fashion so that progressively larger numbers of mid-level and lower-level workers can work in teams with higher-level health care workers. All of these workers need to be well supervised and paid a salary that is commensurate with their level of training and workload to overcome the endemic apathy, absenteeism, and high turnover. Such a PHC system will require regular contact with all households and more frequent visits to those households with greater health needs.

Localization (that is, the decentralization of funds and authority over the use of those funds for PHC) will be required to revitalize the PHC system and to ensure that every locality has the funds it needs to provide essential services. The local entities that receive these funds will need to have the capacity to speak and act on behalf of the communities they serve. Giving citizens a stronger voice in their health services will be essential for improving the quality of PHC.

### 4. Creation of a Strong Professionalized Cadre of Community Health Workers

A strong professionalized cadre of well-trained, full-time CHWs will be needed who can provide a broad array of PHC services beyond maternal and child health, are well supervised, have the needed logistical support, and are closely integrated into the PHC system. CHWs should no longer be considered an underfunded afterthought but rather the foundation of the health system. Globally, CHWs are at the dawn of a new era,^19^ and, in fact, in many countries, they are now leading the way to “Health for All” by achieving high levels of service coverage that are impossible to obtain through facility-based services alone, as Bangladesh has already done so well for reproductive and child health services.^20^ Such a cadre can also provide effective health promotion for control of noncommunicable diseases including hypertension, manage many acute illnesses, and identify those in need of higher levels of care. They also have the potential to register vital events and serve as the “eyes and ears” for the early detection of outbreaks of infectious diseases. Given the high prevalence of hypertension in the Bangladeshi population (around 20% of adults),^21^ the simplicity of identification of cases through home visits from CHWs, and the simplicity and low cost of treatment of most cases, the potential of CHWs to detect and treat hypertension is one of the most exciting opportunities for health improvement in Bangladesh for the foreseeable future.^22^

As one of us (AMRC) has put it^23^:

*In the long-term interests of the health care sector of the country [of Bangladesh], we believe CHWs should be systematically trained and integrated within the health system. We would like to see that they are recognized, at long last, as valued health professionals, something they are, and are paid as such too. This, for Bangladesh, is well within the realm of what is possible. Such choices should not be viewed as second-best solutions for the poor but the best bet for health, and for addressing this [COVID] pandemic and others which are likely to come. We urge that we build on these tested local strengths as a priority rather than aiming to replicate high-cost medical interventions with limited, and in some instances, unproven, prospects of efficacy.*

### 5. Continuation and Further Enhancement of the Culture of Research and Evidence in Health Care

Bangladesh has developed for its health sector a “culture of research and evidence.”^24^ icddr,b, (formerly the International Centre for Diarrhoeal Disease and Research, Bangladesh), and BRAC, among many others, have made enormous contributions by conducting real-world research related to health care delivery, with the learnings applied through iterative implementation. The vital role played early on by Matlab (icddrb’s rural real-world laboratory) in developing the model of maternal and child health and family planning services that was scaled up nationally is well known.^25^

There will be a need for various sites around the country where innovations in health services can be implemented and tested to provide a more scientific and rigorous approach to assessing the effectiveness of innovations in service delivery. The government will need to give these sites the freedom and the money to modify government rules, regulations, and practices. Innovations need to begin at the local level and gradually scale up with monitoring and evaluation at each level of scale, using the science of implementation. The government runs several institutions whose primary task is research, but there are questions as to the relevance and usefulness of their work.

### 6. Citizen Watchdog and Strong Civil Society Leadership

A vibrant, informed civil society (that includes the NGO community) will be essential for Bangladesh to make progress in all these areas. This calls for a strong role for Bangladesh Health Watch and similar organizations that can take a dispassionate view of the health needs of the country, the drawbacks of the health care delivery system, and the steps that need to be taken to address them. This will also require the emergence of strong leaders who can inspire and influence others in these efforts. Making government expenditures transparent and holding the government accountable for these funds will be critical.

## CONCLUSIONS

Throughout the world, good health is routinely put at the top of the list of individuals’ priorities for their own well-being. Health, like education, is among the basic capabilities that give value to human life and that create human capital, one of society’s basic building blocks.^26^ The value that people place on health is also evidenced in many other ways, not the least of which is the vast sums spent in highly developed countries on health care. In the United States in 2020, for example, per capita spending on the health sector was 20% of the national GDP and amounted to US$12,530 per capita.^27^ One can anticipate that Bangladesh will follow a similar path as its economy grows. The overarching question is whether the money spent in Bangladesh over the next 50 years will be used in a way that will bring the greatest benefits possible to the health of the population and particularly to the poorest and most vulnerable members of its society.

The Universal Declaration of Human Rights, which is the United Nations’ moral charter and whose 75th anniversary is being celebrated in 2023, resoundingly affirms that “Everyone has the right to life, liberty and security of person,” that “Everyone has the right to a standard of living adequate for the health and well-being of himself and his family, … including medical care,” and “Motherhood and childhood are entitled to special care and assistance.”^28^ The “right to life” includes the universal right to accessible basic health care services that are effective for preventing and treating serious conditions.

The 1978 Declaration of Alma-Ata called for the achievement of “Health for All” by the year 2000—a level of health that will permit people to lead a socially and economically productive life.^29^ It also affirmed that “[p]rimary health care is the key to attaining this target as part of development in the spirit of social justice.”^29^ The Declaration of Alma-Alta also reminds us that “[t]he people have the right and duty to participate individually and collectively in the planning and implementation of their health care.”^29^ CEA Winslow in 1920 offered his now-classic definition of public health as^30^:

*the science and art of preventing disease, prolonging life, and promoting physical health and efficiency through organized community efforts … which will ensure to every individual in the community a standard of living adequate for the maintenance of health.*

Bangladesh has inspired the rest of the world over the past 50 years because of its effective implementation of the science and art of public health—saving lives and overcoming poverty through organized community efforts, thereby bringing dignity and hope to millions of people. The closing remark in this newly released book proclaims that^31^:

*The government must play a bold role and lend a hand towards the transformation of the health system. That is, the government needs to have courage and full commitment.*

Only through this, but within a pluralistic health system that incorporates the full spirit and participation of citizens and local communities, will such efforts succeed in providing “Health for All.” CHWs reaching every household on a regular basis are the core legacy of Bangladesh’s health advances for the past 50 years. Maintaining and building on this will enable Bangladesh to reach “Health for All” sooner rather than later.

## Notes

**Peer Reviewed**

**First published online: **January 18, 2024.
